# Supplementary material for: Protein interaction network analysis of mTOR signaling reveals modular organization
Source: J Biol Chem. 2023 Sep 21;299(11):105271. doi: 10.1016/j.jbc.2023.105271 (PMC10594569; doi:10.1016/j.jbc.2023.105271)
Supplement: Supporting Figures S1–S8 and Tables S1 [file mmc1.pdf]

Supplemental Material

for:

Multiplex network analysis of protein interactions mediating TOR signal transduction

Authors: Devin Wehle[1,2], Carter S. Bass[2], Josef Šulc[2], Ghayda Mirzaa[2,3,4], Stephen E.P. Smith[1,2,3]

Supplement contains:

Figures S1-S8

Table S1

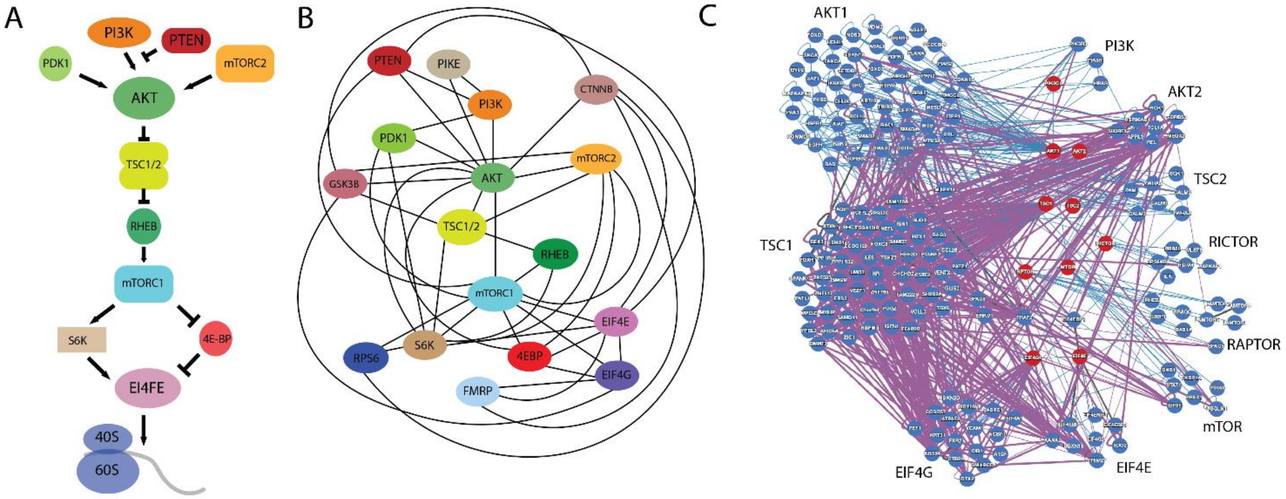

Figure S1: Linear vs. Network modeling of mTOR signal transduction. A) A linear model highlights how each node of the mTOR network affects the next in a simple, organized "cascade". Arrows indicate activation, lines indicate inhibition. B) Known protein-protein interactions in human or mouse cell lines among members of the mTOR signal transduction network, as listed in BioGrid and String databases. The nodes shown are the proteins selected for inclusion in the mTOR QMI panel, lines indicate a documented physical interaction. C) Protein-protein interactions in the Human Reference Interactome database (Luck et al., 2020), based on yeast 2-hybrid screening in immortalized human cells. Red nodes are queried members of the mTOR linear pathway, blue nodes are first-degree interactors, and edges indicate all documented binary interactions between the protein nodes shown. Red nodes are arranged by pathway hierarchy, blue nodes are clustered by interaction partner as labeled.

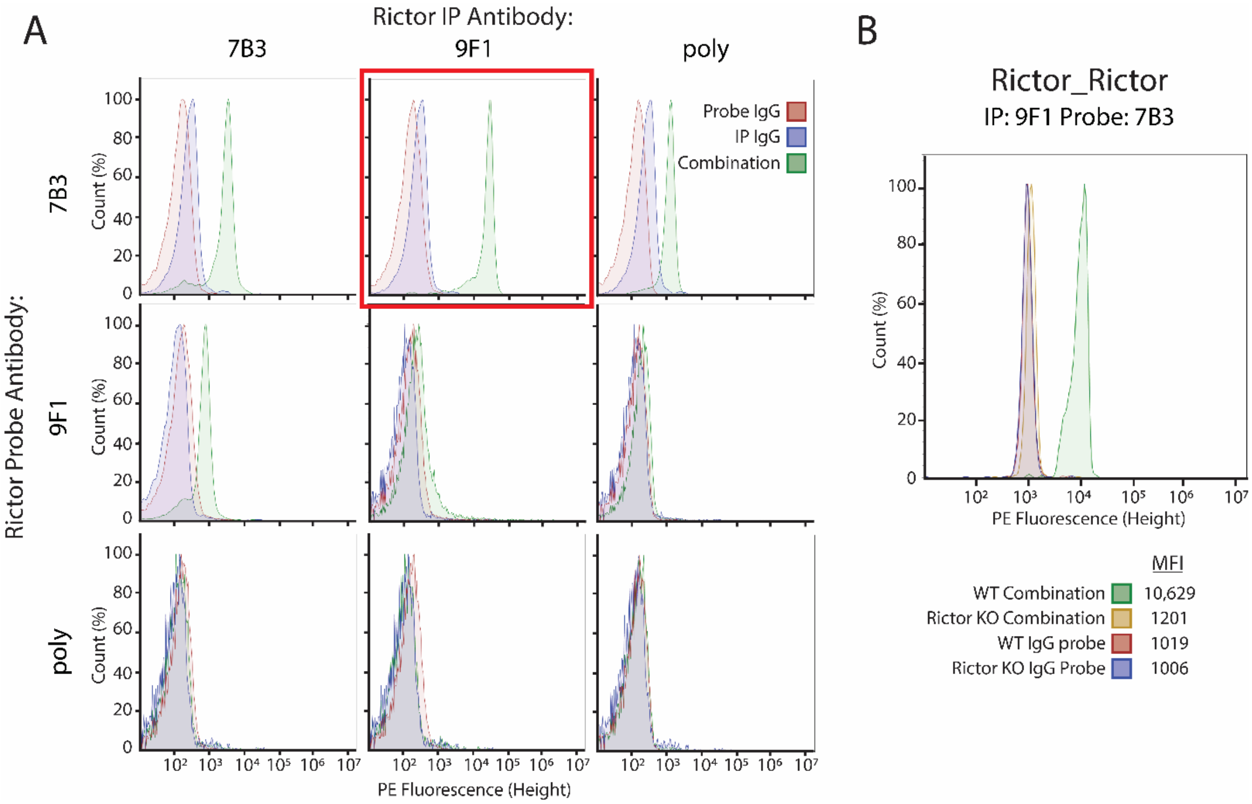

Figure S2. Specificity validation of QMI antibody pairs in mouse. A) Several candidate antibodies were bead-coupled (columns) and biotinylated (rows), and all IP-probe combinations were tested. Histograms for each IP_probe pair (green) were compared to IgG_probe (red) and IP_IgG (blue) controls, and the pair that produced the strongest signal over background was selected (7B3_9F1). B) Specificity was confirmed by comparing wildtype brain lysate to brain lysate from a KO mouse (yellow), which overlapped with IgG controls (red, blue).

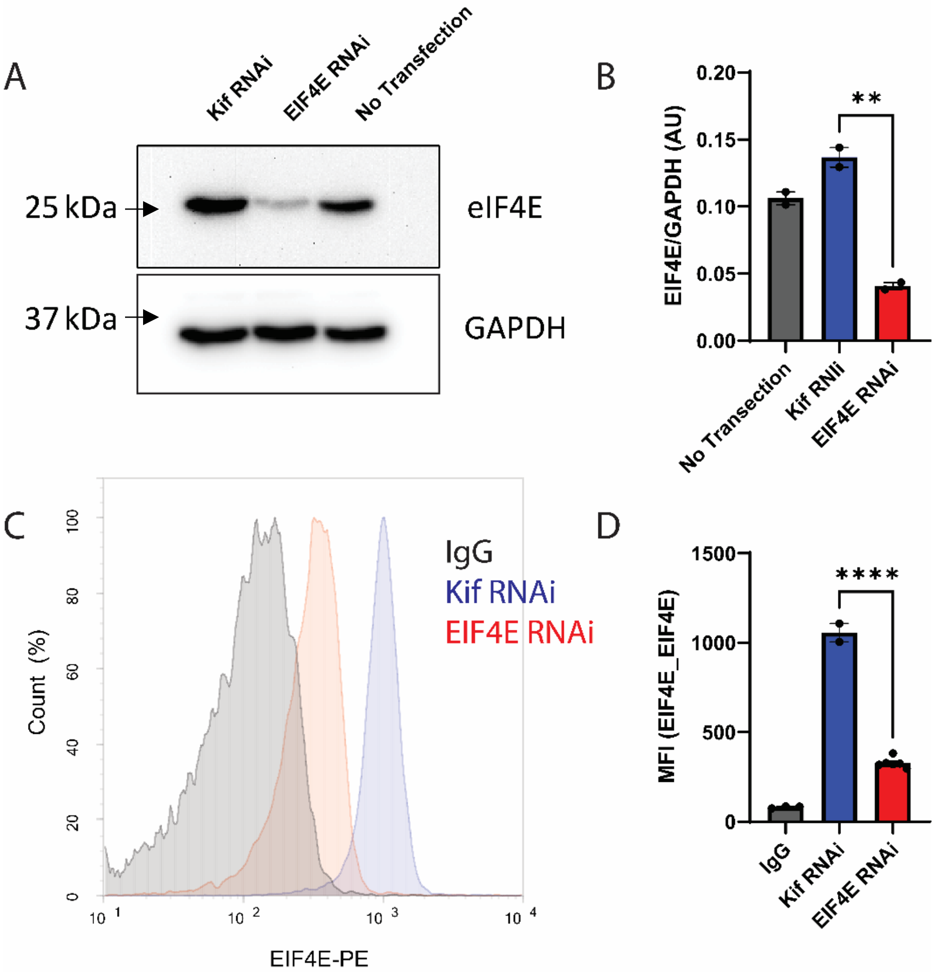

Figure S3: Specificity validation of QMI antibody pairs in human (A) Western blots from HEK 293 cells treated with RNAi to knockdown EIF4E. (B) Quantification of A, normalized to GAPDH levels. N=2 independent experiments, analyzed in duplicate. ** indicates significantly different from the KIF positive control by ANOVA followed by Bonferroni-corrected post-hoc testing, $p<0.05$. (C) Human specificity was confirmed by comparing KIF treated 293 cells (blue) to EIF4E RNAi treated 293 cells (red) and IGG precipitated samples (grey). (D) Quantification of the median fluorescent values from C. N=2 independent experiments, analyzed in duplicate. ** indicates significantly different

from the KIF positive control by ANOVA followed by Bonferroni-corrected post-hoc testing, $p<0.05$.

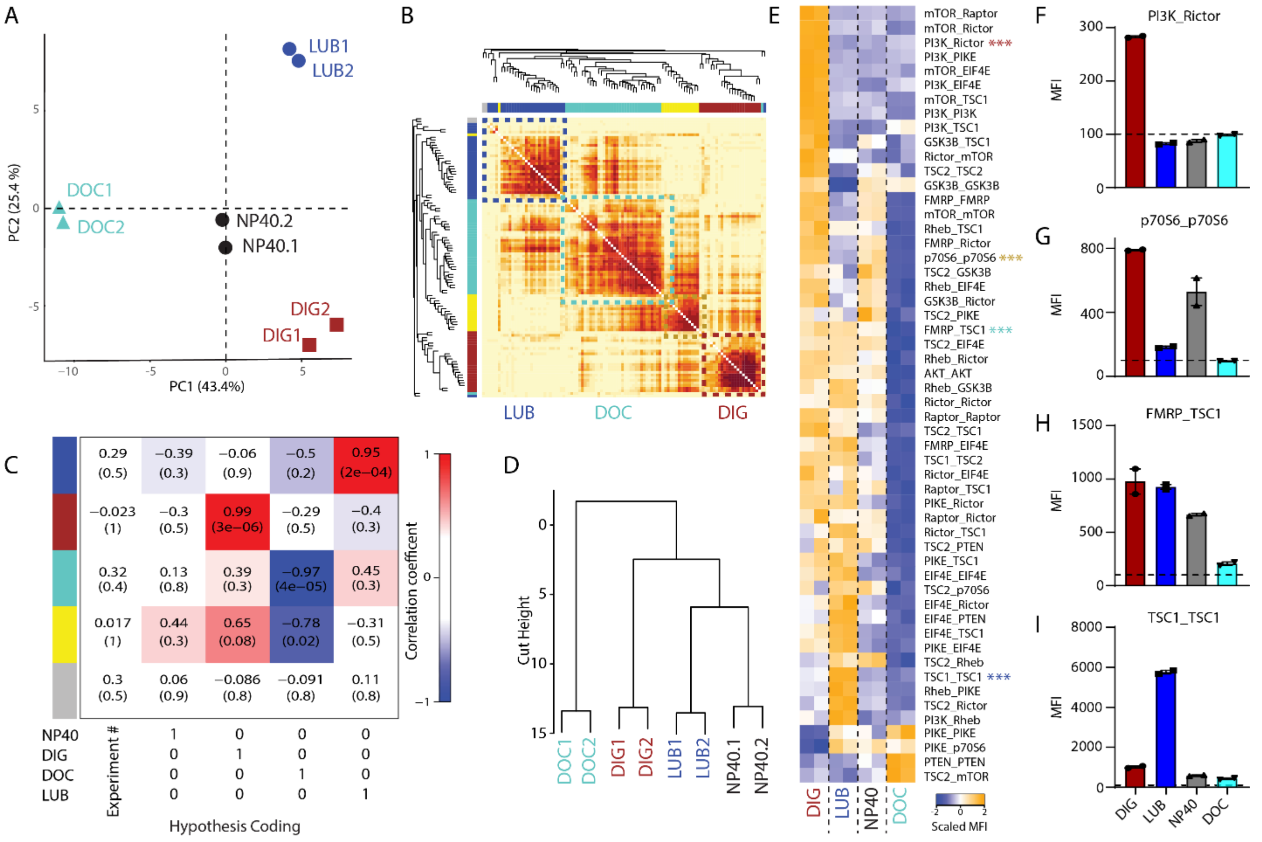

Figure S4: Detergent affects protein complexes detected by QMI (A) Principal component analysis (PCA) of P0 mouse cortical lysates lysed in four different detergents: Deoxycholate (DOC-turquoise), NP-40 (black), Lubrol (LUB-blue), and Digitonin (DIG-red) N=2 per condition. (B). A topological overlap matrix (TOM) reveals four modules of correlated PiSCES; dashed boxes outline each arbitrariliy-colored module. (C). Module-trait table showing the correlation (top number) and the p-value (bottom number) calculated by CNA for each trait. Hypothesis coding table below indicates the binary coding of detergent "traits". (D). Hierarchical clustering of QMI data showing strong correlation between detergent repliates, and seperation of data by detergent (E). Heatmap of the median scaled values of all dynamic interactions that showed significant changes between deteregent conditions. Each box represents a

single interaction measurement from a single experimental replicate; columns correspond to a replicate while rows correspond to an interaction. N=2. (F-I). Bar graphs of the median fluorescent intensity (MFI) from a representative interaction in each module across samples.

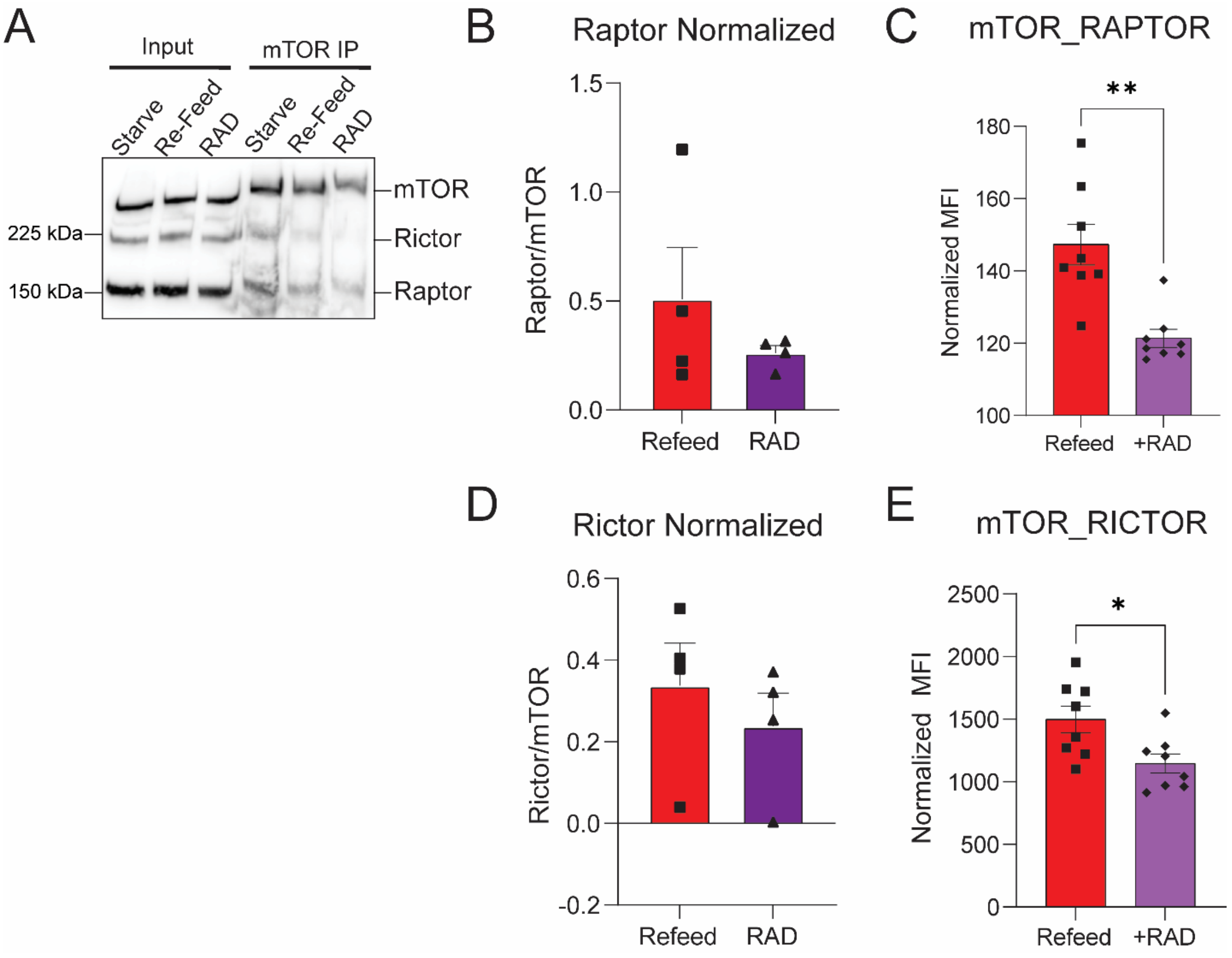

Figure S5: Co-immunoprecipitation of mTOR following serum starvation and RAD001 treatment (A) IP-western blots from 3T3 fibroblasts underwent serum starvation and refeeding protocol with or without 40 nM RAD001 and lysed in 0.3% Chaps buffer. (B) Quantification of Raptor probe from Refed and RAD treated samples normalized to mTOR (N=4). (C) The normalized median fluorescent values of the mTOR_RAPTOR interaction from Figure 2. ** indicates $p<0.01$ by t-test. (D) Quantification of Rictor probe from Refed and RAD treated samples normalized to mTOR (N=4). (C) The normalized median fluorescent values of the mTOR_RICTOR interaction from Figure 2. * indicates $p<0.05$ by t-test.

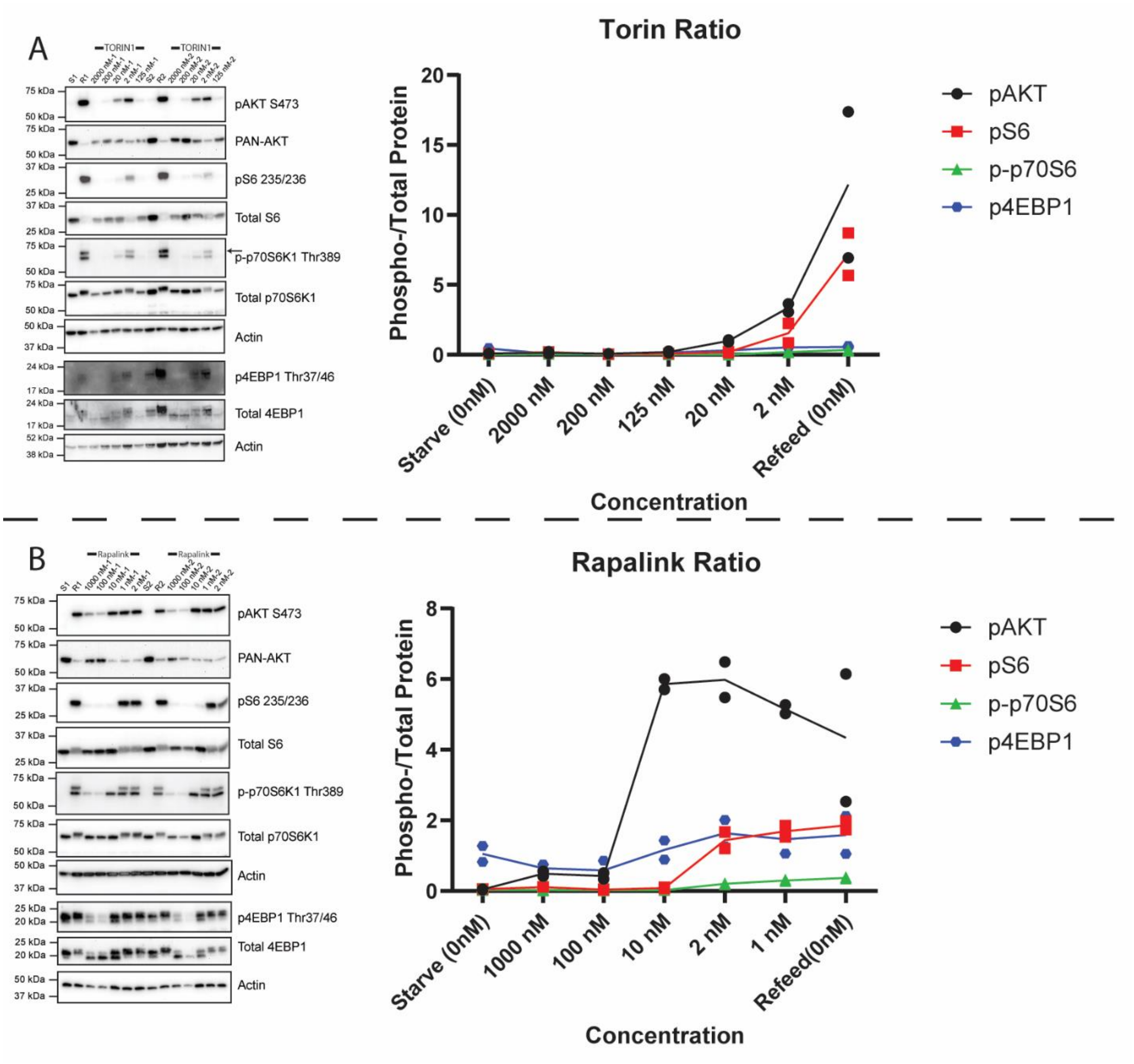

Figure S6: (continued on next page)

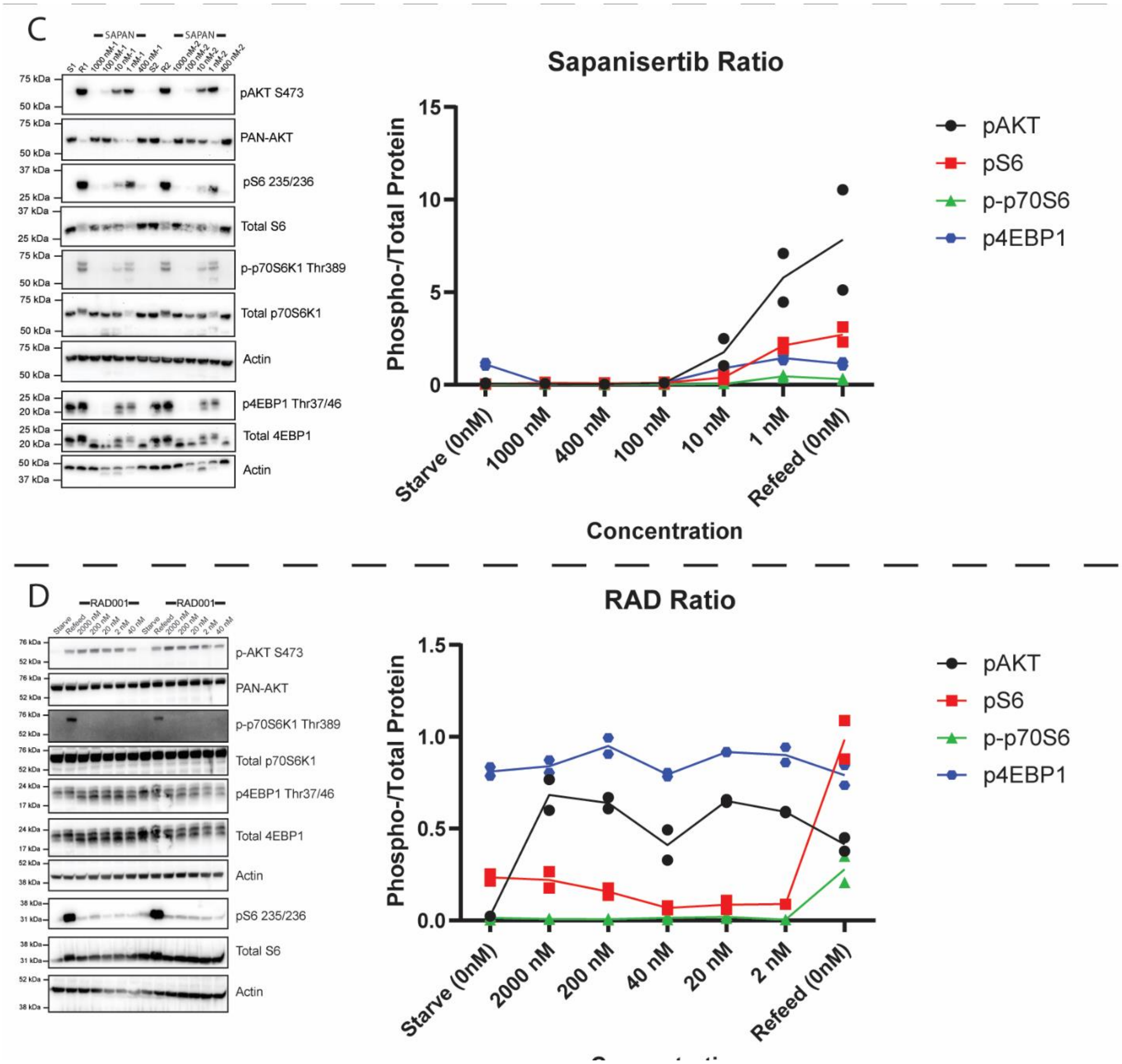

Figure S6: Phosphorylation level curves related to Figure 4. A) Phospho-western blots for AKT (black), S6 (red), p70S6 (green), and 4EBP1 (blue) from a serum-starvation experiment in the presence of various concentrations of TORIN1 along with a graph representing quantification at different concentrations using the ratio of phosphorylated to total protein (N=2). B-D) Similar figures as S4A but for Rapalink, Sapanisertib, and RAD001 respectively.

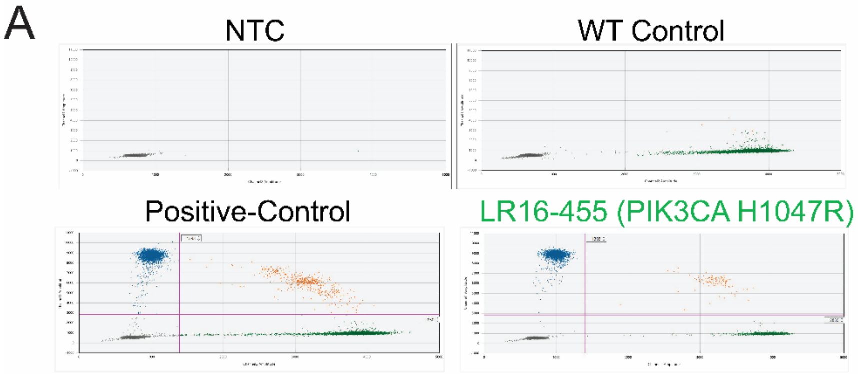

Sample	Date	VAF (%)	VAF Max (%)	VAF Min (%)	Delta CI (%)	Accepted Droplets	Mut Positive	WT Positive	Probeset
NTC	11/29/2021	0.00	0.00	0.00		55721	0	1	H1047R
WT Control	11/29/2021	0.03	0.06	0.00		52789	4	12633	H1047R
Pos Con LR18-349 1702094 (22%)	11/29/2021	24.99	25.78	24.21	24.15	57679	2956	8424	H1047R
LR16-455 1702093	11/29/2021	49.94	51.29	48.60	48.54	61892	2661	2667	H1047R

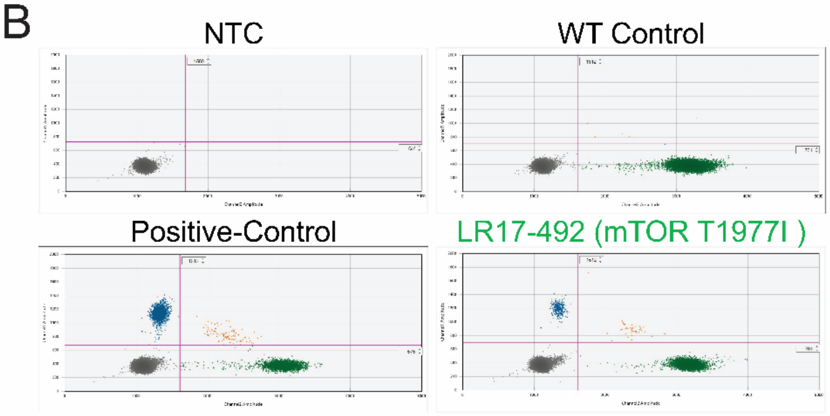

Sample	Date	VAF (%)	VAF Max (%)	VAF Min (%)	Delta CI (%)	Accepted Droplets	Mut Positive	WT Positive	Probeset
NTC	11/9/2021					57496	0	1	T1977I
WT Control	11/9/2021					61929	6	19565	T1977I
LR17-492 1802021 Pos Con (41%)	11/9/2021	39.32	40.66	37.98	37.98	63552	2016	3084	T1977I
LR17 492	11/9/2021	5.15	5.66	4.65	4.65	58556	379	6598	T1977I

Figure S7: Droplet digital PCR of patient derived mutant mTOR PIK3CA and mTOR (A) Droplet digital PCR results for the PIK3CA H1047R line, summarized in a table. (B) Droplet digital PCR results for the mTOR T1977I line, summarized in a table.

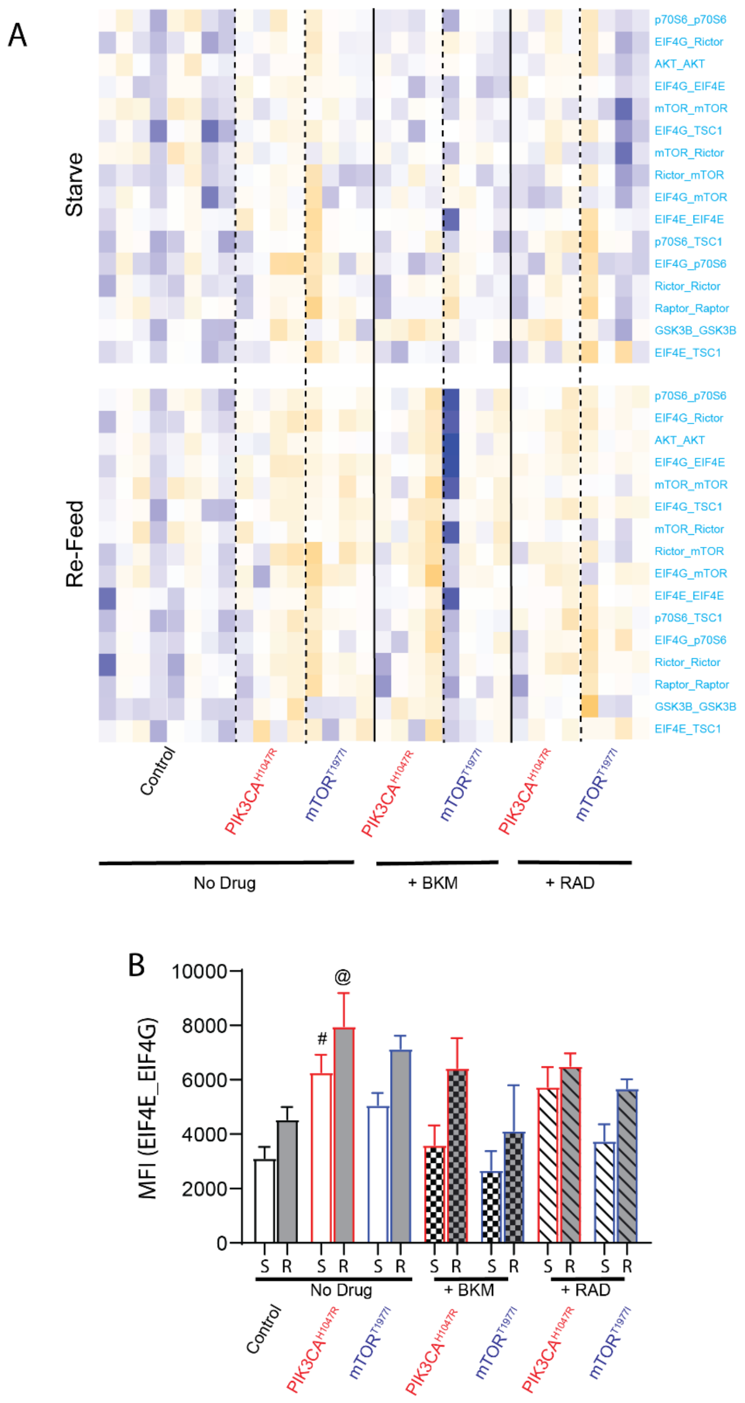

Figure S8: Mutant fibroblast drug inhibition experiment, related to Figure 5. A) Heatmap of the median scaled values of all dynamic PiSCES that showed ANC∩CNA-significant changes. Each box represents a single interaction measurement from a single experimental replicate; columns correspond to an experimental replicate while

rows correspond to an interaction. N = 4-8. B) Median fluorescent intensity (MFI) of EIF4E_EIF4G, the interaction most correlated with the turquoise module, shown for all conditions. * indicates significantly different from starved within-genotype, # significantly different from control-starved, and @ signficantly different from control-re-fed, by ANOVA followed by Bonferroni-corrected post-hoc testing, $p<0.05$. N=4-8

Table S1: Antibody pairs used for QMI

	Target	Uniprot identifier	Uniprot #	IP antibody clone	IP antibody Catalog number	IP antibody Supplier	Probe antibody clone	Probe antibody Catalog number	Probe antibody Supplier	Mouse Validation	Human Validation
1	AKT1	AKT1_MOUSE	P31750	SKB1	05-591	Millipore	B1	sc-5298	Santa Cruz	siRNA	RNAi
2	Beta-Catenin	CTNB1_MOUSE	Q02248	196624	MAB13292	R&D Systems	OTI2H7	NBP2-03615	Novus Biologicals	Mouse KO liver lysate (B6.129-Ctnnb1tm2Kem/KnwJ)	RNAi
3	eIF4E	IF4E_MOUSE	P63073	299910	MAB3228	R&D Systems	Poluclonal	A301-153A	Bethyl	siRNA	RNAi
4	EIF4G	IF4G1_MOUSE	Q6NZJ6	G6899	MA5-14971	ThermoFisher-Scientific	3A10	SAB1403762	Sigma-Aldrich	siRNA	
5	FMRP	FMR1_MOUSE	P35922	Polyclonal	F4055	Sigma-Aldrich	5C2	MMS-5232	Biolegend	Mouse KO brain lystate (B6.129P2-Fmr1tm1Cgr/J)	RNAi
6	GSK3B	GSK3B_MOUSE	Q9WV60	3D6B4	NBP2-52478	Novus Biologicals	3D10	NBP1-47470	Novus Biologicals	Mouse Embryonic fibroblast lysate (GSK3β(-/-); GSK3α(flox/-) (3/4KO)	RNAi
7	mGluR5	GRM5_MOUSE	Q3UVX5	5675	AB5675	Millipore	N75/3	75-115	Neuromab	Plasmid overexpression (Lautz et al 2018)	
8	mTOR	MTOR_MOUSE	Q9JLN9	2ID8.2	05-1592	Millipore	30	sc-517464	Santa Cruz	siRNA	RNAi
9	p70S6K	KS6A1_MOUSE	P18653	215247	MAB8962	R&D Systems	Polyclonal	AF8962	Novus Biologicals	Mouse KO liver lysate (C.129(B6)-Rps6kb1tm1Gtho/NsoJ)	RNAi
10	PI3K	P85A_MOUSE	P26450	U5	MA1-74183	ThermoFisher-Scientific	AB6	05-212	Millipore	Plasmid overexpression (Lautz et al 2018)	Smith et al, 2016
11	PIKE	AGAP2_MOUSE	Q3UHD9	263A	A304-263A	Bethyl	DN8	200-401-DN8	Rockland	X63 Myeloma (Smith et al, 2016)	Smith et al, 2016
12	PTEN	PTEN_MOUSE	O08586	217702	MAB847	Novus Biologicals	6H2.1	04-035	Millipore	Plasmid overexpression	
13	RAPTOR	RPTOR_MOUSE	Q8K4Q0	Polyclonal	42-4000	ThermoFisher-Scientific	514208	MAB5957	Novus Biologicals	siRNA	RNAi
14	Rheb	RHEB_MOUSE	Q921J2	344912	MAB3426	R&D Systems	JG37-12	NBP2-75646	Novus Biologicals	Plasmid overexpression	RNAi
15	RICTOR	RICTR_mouse	Q6QI06	7B3	05-1471	Novus Biologicals	9F1.2	Novus NBP1-51645	Millipore	mouse KO ESC lysate	
16	TSC1	TSC1_MOUSE	Q9EP53	5C8A12	37-0400	ThermoFisher-Scientific	Polyclonal	NB100-2315	Novus Biologicals	Mouse Embryonic fibroblast lysate (TSC1(-/-);)	RNAi
17	TSC2	TSC2_MOUSE	Q61037	614204	MAB40401	R&D Systems	SC05-59	NBP2-67552	Novus Biologicals	Plasmid overexpression	RNAi
